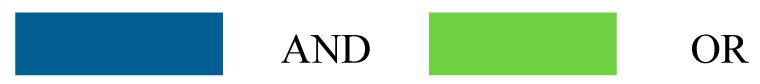# In-Flight Estimation of Center of Gravity Position Using All-Accelerometers

**DOI:** 10.3390/s140917567

**Published:** 2014-09-19

**Authors:** Yazan Mohammad Al-Rawashdeh, Moustafa Elshafei, Mohammad Fahad Al-Malki

**Affiliations:** 1 Department of Systems Engineering, King Fahd University of Petroleum and Minerals, Dhahran 31261, Saudi Arabia; E-Mail: Shafei@mit.edu or elshafei@kfupm.edu.sa; 2 Technical & Industrial Services Co., Dammam 31493, Saudi Arabia; E-Mail: mfmalki@ieee.org

**Keywords:** in-flight, center of gravity determination, all-accelerometers, IMU

## Abstract

Changing the position of the Center of Gravity (CoG) for an aerial vehicle is a challenging part in navigation, and control of such vehicles. In this paper, an all-accelerometers-based inertial measurement unit is presented, with a proposed method for on-line estimation of the position of the CoG. The accelerometers' readings are used to find and correct the vehicle's angular velocity and acceleration using an Extended Kalman Filter. Next, the accelerometers' readings along with the estimated angular velocity and acceleration are used in an identification scheme to estimate the position of the CoG and the vehicle's linear acceleration. The estimated position of the CoG and motion measurements can then be used to update the control rules to achieve better trim conditions for the air vehicle.

## 1. Introduction

The dynamic equations of an aircraft vehicle are normally derived under the assumption of known and stationary Center of Gravity (CoG). Variations in loads due to picking up/dropping off loads or consuming fuel could result in a change in both the vehicle's mass and position of CoG. This immigration in the position of CoG introduces undesirable couplings in the flight dynamics [1]. This dynamic coupling may appear in the angular, as well as the linear, acceleration and in the lateral and longitudinal motions [2]. According to United States Federal Aviation Administration (FAA) [3], a CoG limits envelope is determined for each aircraft, within which a safe and good flight conditions can be maintained even under CoG position changes. Different approaches to estimate the position of CoG have been reported in literatures. For example, in [2], an adaptive weighted data fusion, based on optimal weight distribution, and an identification technique based on neural network were utilized to improve the effectiveness of such estimation. In [1], the change in CoG position was modeled to find its effect on an aircraft under heavy load airdrop. In [4], both static and dynamic measurements were used to estimate the position of CoG of a helicopter on ground and in-flight respectively. The dynamic approach of [4] depends on finding the modal frequencies by solving an eigenvalue problem, where any change in a certain modal frequency will be an indication of a CoG position. The change in the position of the CoG can then be determined through monitoring the affected frequencies. Changing position of CoG in helicopters was also the main concern of [5], in which an estimation algorithm, based on Extended Kalman Filter (EKF), consists of a rigid body aircraft motion feedback and an internal model of the helicopter. This estimation algorithm was successful in estimating the CoG position within 1 s and its weight within 10 s provided that sufficient motion is present to ensure the observability of the parameters. Different ways of finding CoG position and the moment of inertia of a spacecraft on the ground were also reported in [6].

Having an estimate of CoG position, it can then be transferred to a vehicle management system, such as Active Center of Gravity, that could transfer fuel among the fuel tanks to adjust the position of CoG as required during flight [2], or to update the control approach. In [7], different controllers, namely linear Proportional-Derivative controller (PD), feedback linearization, and adaptive controllers, were tested in controlling a Quadrotor subjected to various disturbances including the change in CoG position where the first two controllers failed to achieve the desired responses.

In both [1] and [2], the estimation of the CoG position depends on the position and mass of the individual objects causing the variations in the mass and CoG, such as cargo and passengers. This knowledge, if possible to have, may lack accuracy and could cause poor estimation accuracy in both the mass and CoG position of the aircraft.

In [5], a dynamic model of the helicopter was used along its weight and balance, *i.e.*, CoG position, to build an EKF that was used to estimate both the gross weight and CoG location of the helicopter.

Unlike the previously mentioned methods, the proposed Inertial Measurement Unit (IMU) herein does not need the aircraft dynamic model. As such, it can be used in a broader range of applications.

In [8], a spacecraft center of mass was estimated online using multi-accelerometers under the assumption of zero linear acceleration when the spacecraft is in steady mode. Under such particular condition, the kinematic equations are very much simplified and the position of the CoG can be estimated using a recursive least squares method.

In this paper, a new approach to estimate the CoG position based on an all-accelerometers IMU, as proposed in [9], is evaluated. In [9], the accelerometers are arranged in two or more rings. Two alternative ring versions will be analyzed, simulated, and evaluated. This paper is organized as follows: Section 2 contains the description of the proposed IMUs. Section 3 contains the mathematical derivation. Section 4 contains the simulation results and discussion. A summary is presented in the last section.

## 2. IMU Description

The feasibility of designing all-accelerometers based IMU to compute the linear/angular accelerations, and the angular velocity of a rigid body was investigated in [10]. It is possible to use different number of accelerometers to design all-accelerometers IMU, where the issue of possible singularity must be taken into consideration when finding the optimum number of linear accelerometers arranged in a specific structure [11]. The accelerometers' measurements can be used to determine the angular velocity/acceleration and the linear acceleration of a rigid body using different approaches, such as simple matrix operation [12], or by using filters such as Unscented Kalman Filter (UKF) [13]. It is worth mentioning that IMUs, based on both rate gyros and linear accelerometers, are still under research, see [14].

Using linear accelerometers in certain configurations enables finding the angular acceleration of the body when their outputs are connected in the differential mode. One of those configurations is the diamond configuration by which three pairs of linear accelerometers are separated equally about a point in three perpendicular directions, *i.e.*, one pair per axis. The differential output can then be fed into a Kalman Filter (KF) or the like to estimate the body angular velocities from the noisy linear accelerometers' measurements. Redundant sensors are used to consolidate the accelerometers' measurements. In the proposed IMU found in [9], only two pairs of linear tri-axial accelerometers were used in the *Y* and *Z* directions which make a total number of 12 accelerometers. The available accelerometers' channels in each tri-axial accelerometer can be used for fault detection and isolation [9].

**Figure 1:**
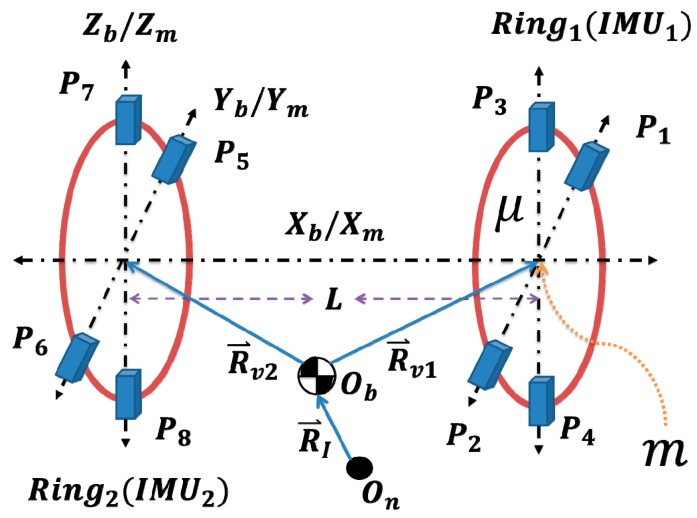
Two-Ring approach based on the proposed IMU using 12-accelerometer/Ring version [9].

The basic form of the proposed IMU, as found in [9], is shown in Figure 1, in which accelerometers are arranged in the form of two rings. The proposed IMU helps in improving navigation and control of aerial vehicles, and more particularly in tracking the changes in the position of CoG of a moving vehicle due to fuel consumption or changes in its payload. Redundancy is available at the IMU intra ring level and at the inter rings level to increase the reliability of such instrument. The distribution of these rings can be made flexible to overcome possible installation constraints in real vehicles.

In Figure 1, assume all the accelerometers to be placed symmetrically around a point *m* at a distance μ where *P_i_* is a tri-axial linear accelerometer's position, *O_b_* is the position of CoG, *O_n_* is the origin of the inertial coordinate system, *R_I_* is the vector from inertial frame origin *O_n_* to CoG, and *R_vj_* is the vector from the CoG *O_b_* to origin of Ring (*j*), where *j* = 1, 2, 3, ... The Ring's coordinate system (*mX_m_Y_m_Z_m_*) is assumed to be perfectly aligned with the vehicle's body coordinate system(*O_b_X_b_Y_b_Z_b_*).

The first step in estimating the position of the CoG, using the proposed IMUs, is the derivation of the body angular velocities from the accelerometers' measurements. Several methods were proposed for estimating the angular velocities using all-accelerometers systems, see [1,2] for example.

In the following section, the mathematical derivation of the angular velocities, linear/angular accelerations, and CoG position estimation for two versions of the proposed IMU is presented briefly, where the first version uses 12 accelerometers per Ring, while the second version uses 18 accelerometers per Ring.

## 3. Mathematical Derivation

**Figure 2:**
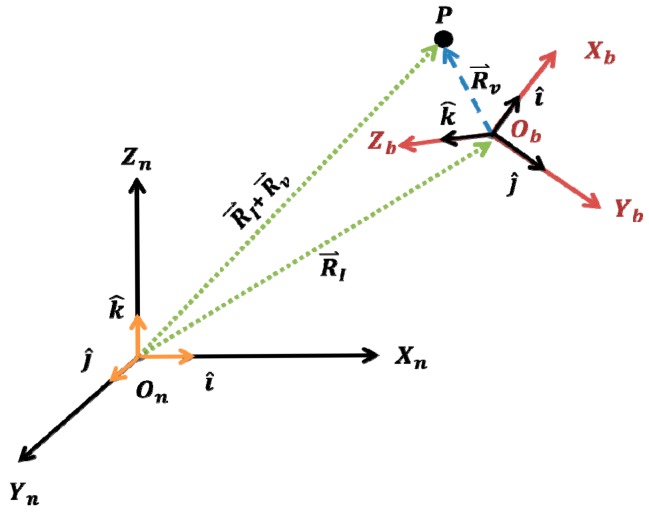
Inertial (*O_n_X_n_Y_n_Z_n_*) and body (*O_b_X_b_Y_b_Z_b_*) frames.

Adopting a flat non-rotating Earth model, the measurements of an accelerometer located at point *P* are given by, see Figure 2:

(1) $${\vec{A}}_{I}={\ddot{\vec{R}}}_{I}+\dot{\vec{\Omega }}\times {\vec{R}}_{v}+{\ddot{\vec{R}}}_{v}+2\vec{\Omega }\times {\dot{\vec{R}}}_{v}+\vec{\Omega }\times (\vec{\Omega }\times {\vec{R}}_{v})-\vec{g}$$

where:

*A⃑_I_*, is the inertial acceleration of arbitrary point *P* measured in body coordinate system (*O_b_X_b_Y_b_Z_b_*)
${\ddot{\vec{R}}}_{I}$, is the linear acceleration of the origin of the body coordinate system with respect to inertial space (*O_n_X_n_Y_n_Z_n_*).
*R⃑_v_*, is the vector from the origin of the body coordinate system (*O_b_*) to point *P*.
Ω⃑, is the angular velocity of the body system.
$\dot{\vec{\Omega }}$, is the angular acceleration of the body system.
×, denotes the cross product between two vectors.
*g⃑*,is the gravitational acceleration.

Now, if the CoG is stationary, *i.e.*, ${\ddot{\vec{R}}}_{v}={\dot{\vec{R}}}_{v}=\vec{0}$, then the accelerometer's measurements at a point *P* is given by:

(2) $${\vec{A}}_{I}={\ddot{\vec{R}}}_{I}+\dot{\vec{\Omega }}\times {\vec{R}}_{v}+\vec{\Omega }\times (\vec{\Omega }\times {\vec{R}}_{v})-\vec{g}$$

### 3.1. Estimating the Angular Velocities and Accelerations

Referring to Figure 1, the accelerometers' measurements at points *P1*, *P2*, *P3*, and *P4* can be expressed as:

(3) $$\begin{array}{c} {\vec{A}}_{1}={\ddot{\vec{R}}}_{I}+{\ddot{\vec{R}}}_{v1}+\dot{\vec{\Omega }}\times \left({\vec{R}}_{v1}+µ\vec{J}\right)+2\vec{\Omega }\times {\dot{\vec{R}}}_{v1}+\vec{\Omega }\times \left(\vec{\Omega }\times \left({\vec{R}}_{v1}+µ\vec{J}\right)\right)-\vec{g}\\ {\vec{A}}_{2}={\ddot{\vec{R}}}_{I}+{\ddot{\vec{R}}}_{v1}+\dot{\vec{\Omega }}\times \left({\vec{R}}_{v1}-µ\vec{J}\right)+2\vec{\Omega }\times {\dot{\vec{R}}}_{v1}+\vec{\Omega }\times \left(\vec{\Omega }\times \left({\vec{R}}_{v1}+µ\vec{J}\right)\right)-\vec{g}\\ {\vec{A}}_{3}={\ddot{\vec{R}}}_{I}+{\ddot{\vec{R}}}_{v1}+\dot{\vec{\Omega }}\times \left({\vec{R}}_{v1}+µ\vec{J}\right)+2\vec{\Omega }\times {\dot{\vec{R}}}_{v1}+\vec{\Omega }\times \left(\vec{\Omega }\times \left({\vec{R}}_{v1}+µ\vec{k}\right)\right)-\vec{g}\\ {\vec{A}}_{4}={\ddot{\vec{R}}}_{I}+{\ddot{\vec{R}}}_{v1}+\dot{\vec{\Omega }}\times \left({\vec{R}}_{v1}+µ\vec{J}\right)+2\vec{\Omega }\times {\dot{\vec{R}}}_{v1}+\vec{\Omega }\times \left(\vec{\Omega }\times \left({\vec{R}}_{v1}+µ\vec{k}\right)\right)-\vec{g} \end{array}$$

Or, by using the skew-symmetric matrix notation instead of the cross product, the accelerometer's measurements at *P1* can be given as:

(4) $$\begin{array}{c} \left[\begin{array}{c} A_{1x}\\ A_{1y}\\ A_{1z} \end{array}\right]=\left[\begin{array}{c} a_{x}\\ a_{y}\\ a_{z} \end{array}\right]+\left[\begin{array}{c} {\ddot{r}}_{v1x}\\ {\ddot{r}}_{v1y}\\ {\ddot{r}}_{v1z} \end{array}\right]+2\left[\begin{array}{ccc} 0 & -\Omega_{z} & \Omega_{y}\\ \Omega_{z} & 0 & -\Omega_{x}\\ -\Omega_{y} & \Omega_{x} & 0 \end{array}\right]\left[\begin{array}{c} {\dot{r}}_{v1x}\\ {\dot{r}}_{v1y}\\ {\dot{r}}_{v1z} \end{array}\right]+\left[\begin{array}{ccc} 0 & -{\dot{\Omega }}_{z} & {\dot{\Omega }}_{y}\\ {\dot{\Omega }}_{z} & 0 & -{\dot{\Omega }}_{x}\\ -{\dot{\Omega }}_{y} & {\dot{\Omega }}_{x} & 0 \end{array}\right]\left[\begin{array}{c} r_{v1x}\\ r_{v1y}+µ\\ r_{v1z} \end{array}\right]\\ +\left[\begin{array}{ccc} -\Omega_{z}^{2}-\Omega_{y}^{2} & \Omega_{x}\Omega_{y} & \Omega_{x}\Omega_{z}\\ \Omega_{x}\Omega_{y} & -\Omega_{z}^{2}-\Omega_{x}^{2} & \Omega_{z}\Omega_{y}\\ \Omega_{x}\Omega_{z} & \Omega_{z}\Omega_{y} & -\Omega_{y}^{2}-\Omega_{x}^{2} \end{array}\right]\left[\begin{array}{c} r_{v1x}\\ r_{v1y}+µ\\ r_{v1z} \end{array}\right]-\vec{g} \end{array}$$

where, *A⃑1* = [*A_1x_,A_1y_* *A_1z_*]*^T^*, ${\ddot{\vec{R}}}_{I}={\left[a_{x},a_{y},a_{z}\right]}^{T}$, *R⃑_v_*_1_ = [*r_v_*_1_*_x_*,*r_v_*_1_*_z_*,*r_v_*_1_*_z_*]*^T^* and Ω⃑ = [Ω*_x_*, Ω*_y_*, Ω*_z_*]*^T^*. Similarly, the acceleration as measured at *P2* is given by:

(5) $$\begin{array}{c} \left[\begin{array}{c} A_{2x}\\ A_{2y}\\ A_{2z} \end{array}\right]=\left[\begin{array}{c} a_{x}\\ a_{y}\\ a_{z} \end{array}\right]+\left[\begin{array}{c} {\ddot{r}}_{v1x}\\ {\ddot{r}}_{v1y}\\ {\ddot{r}}_{v1z} \end{array}\right]+2\left[\begin{array}{ccc} 0 & -\Omega_{z} & \Omega_{y}\\ \Omega_{z} & 0 & -\Omega_{x}\\ -\Omega_{y} & \Omega_{x} & 0 \end{array}\right]\left[\begin{array}{c} {\dot{r}}_{v1x}\\ {\dot{r}}_{v1y}\\ {\dot{r}}_{v1z} \end{array}\right]+\left[\begin{array}{ccc} 0 & -{\dot{\Omega }}_{z} & {\dot{\Omega }}_{y}\\ {\dot{\Omega }}_{z} & 0 & -{\dot{\Omega }}_{x}\\ -{\dot{\Omega }}_{y} & {\dot{\Omega }}_{x} & 0 \end{array}\right]\left[\begin{array}{c} r_{v1x}\\ r_{v1y}-µ\\ r_{v1z} \end{array}\right]\\ +\left[\begin{array}{ccc} -\Omega_{z}^{2}-\Omega_{y}^{2} & \Omega_{x}\Omega_{y} & \Omega_{x}\Omega_{z}\\ \Omega_{x}\Omega_{y} & -\Omega_{z}^{2}-\Omega_{x}^{2} & \Omega_{z}\Omega_{y}\\ \Omega_{x}\Omega_{z} & \Omega_{z}\Omega_{y} & -\Omega_{y}^{2}-\Omega_{x}^{2} \end{array}\right]\left[\begin{array}{c} r_{v1x}\\ r_{1vy}-µ\\ r_{v1z} \end{array}\right]-\vec{g} \end{array}$$

The differential output of the accelerometers at points *P1* and *P2* is given by:

(6) $$\left[\begin{array}{c} A_{1x}\\ A_{1y}\\ A_{1z} \end{array}\right]-\left[\begin{array}{c} A_{2x}\\ A_{2y}\\ A_{2z} \end{array}\right]=\left[\begin{array}{ccc} 0 & -{\dot{\Omega }}_{z} & {\dot{\Omega }}_{y}\\ {\dot{\Omega }}_{z} & 0 & -{\dot{\Omega }}_{x}\\ -{\dot{\Omega }}_{y} & {\dot{\Omega }}_{x} & 0 \end{array}\right]\left[\begin{array}{c} 0\\ 2µ\\ 0 \end{array}\right]+\left[\begin{array}{ccc} -\Omega_{z}^{2}-\Omega_{y}^{2} & \Omega_{x}\Omega_{y} & \Omega_{x}\Omega_{z}\\ \Omega_{x}\Omega_{y} & -\Omega_{z}^{2}-\Omega_{x}^{2} & \Omega_{z}\Omega_{y}\\ \Omega_{x}\Omega_{z} & \Omega_{z}\Omega_{y} & -\Omega_{y}^{2}-\Omega_{x}^{2} \end{array}\right]\left[\begin{array}{c} 0\\ 2µ\\ 0 \end{array}\right]$$

Similarly, the differential output of the accelerometers at points *P3* and *P4* is given by:

(7) $$\left[\begin{array}{c} A_{3x}\\ A_{3y}\\ A_{3z} \end{array}\right]-\left[\begin{array}{c} A_{4x}\\ A_{4y}\\ A_{4z} \end{array}\right]=\left[\begin{array}{ccc} 0 & -{\dot{\Omega }}_{z} & {\dot{\Omega }}_{y}\\ {\dot{\Omega }}_{z} & 0 & -{\dot{\Omega }}_{x}\\ -{\dot{\Omega }}_{y} & {\dot{\Omega }}_{x} & 0 \end{array}\right]\left[\begin{array}{c} 0\\ 0\\ 2µ \end{array}\right]+\left[\begin{array}{ccc} -\Omega_{z}^{2}-\Omega_{y}^{2} & \Omega_{x}\Omega_{y} & \Omega_{x}\Omega_{z}\\ \Omega_{x}\Omega_{y} & -\Omega_{z}^{2}-\Omega_{x}^{2} & \Omega_{z}\Omega_{y}\\ \Omega_{x}\Omega_{z} & \Omega_{z}\Omega_{y} & -\Omega_{y}^{2}-\Omega_{x}^{2} \end{array}\right]\left[\begin{array}{c} 0\\ 0\\ 2µ \end{array}\right]$$

Similarly, the differential output between points *P1* and *P5* is given by:

(8) $$\left[\begin{array}{c} A_{1x}\\ A_{1y}\\ A_{1z} \end{array}\right]-\left[\begin{array}{c} A_{5x}\\ A_{5y}\\ A_{5z} \end{array}\right]=\left[\begin{array}{ccc} 0 & -{\dot{\Omega }}_{z} & {\dot{\Omega }}_{y}\\ {\dot{\Omega }}_{z} & 0 & -{\dot{\Omega }}_{x}\\ -{\dot{\Omega }}_{y} & {\dot{\Omega }}_{x} & 0 \end{array}\right]\left[\begin{array}{c} L\\ 0\\ 0 \end{array}\right]+\left[\begin{array}{ccc} -\Omega_{z}^{2}-\Omega_{y}^{2} & \Omega_{x}\Omega_{y} & \Omega_{x}\Omega_{z}\\ \Omega_{x}\Omega_{y} & -\Omega_{z}^{2}-\Omega_{x}^{2} & \Omega_{z}\Omega_{y}\\ \Omega_{x}\Omega_{z} & \Omega_{z}\Omega_{y} & -\Omega_{y}^{2}-\Omega_{x}^{2} \end{array}\right]\left[\begin{array}{c} L\\ 0\\ 0 \end{array}\right]$$

where, (*L*) is the separation between the two Rings along the vehicle's *X*-axis.

The usage of the differential output helps in canceling out the gravity effect when estimating the angular velocities in the proposed IMUs.

Equations (6)–(8) can be stacked as follows:

(9) $$\left[\begin{array}{ccc} \frac{{\vec{A}}_{1}-{\vec{A}}_{5}}{L} & \frac{{\vec{A}}_{1}-{\vec{A}}_{2}}{2µ} & \frac{{\vec{A}}_{3}-{\vec{A}}_{4}}{2µ} \end{array}\right]=\left[\begin{array}{ccc} 0 & -{\dot{\Omega }}_{z} & {\dot{\Omega }}_{y}\\ {\dot{\Omega }}_{z} & 0 & -{\dot{\Omega }}_{x}\\ -{\dot{\Omega }}_{y} & {\dot{\Omega }}_{x} & 0 \end{array}\right]+\left[\begin{array}{ccc} -\Omega_{z}^{2}-\Omega_{y}^{2} & \Omega_{x}\Omega_{y} & \Omega_{x}\Omega_{z}\\ \Omega_{x}\Omega_{y} & -\Omega_{z}^{2}-\Omega_{x}^{2} & \Omega_{z}\Omega_{y}\\ \Omega_{x}\Omega_{z} & \Omega_{z}\Omega_{y} & -\Omega_{y}^{2}-\Omega_{x}^{2} \end{array}\right]$$

where (*A⃑_l_* = [*A_lx_ A_ly_ A_lz_*]*^T^*), and (*l*) is the accelerometer index.

Using Equation (9), the first new set of equations can be obtained as summarized in Table 1. It is worth noting that Equation (9) can be used to derive other measurement models that can be used alternatively in this setup.

The set of equations summarized in Table 1 are used with Ring 1, and it can also be used with Ring 2 by simply replacing (*L*) with (−*L*).

**Figure 3:**
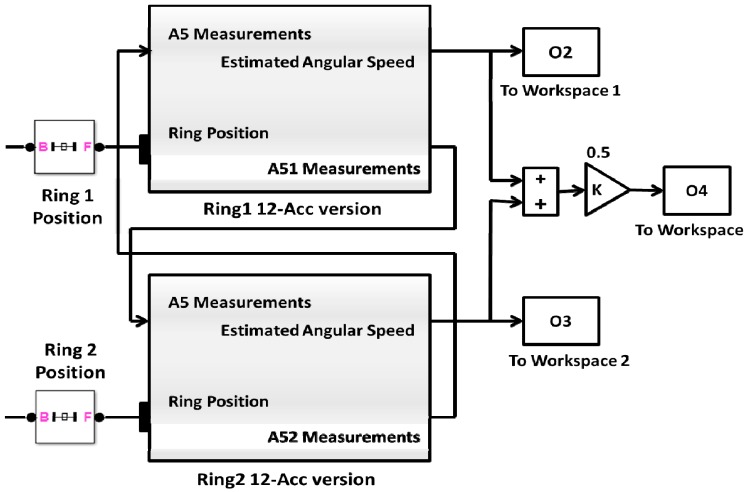
Estimate Angular Velocity using first version of 12-accelerometers/Ring.

**Figure 4:**
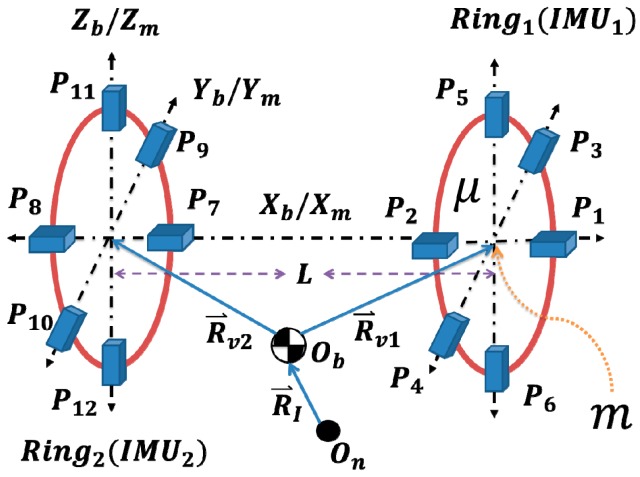
Two-Ring approach, based on the proposed IMU using 18-accelerometer/Ring version.

**Figure 5:**
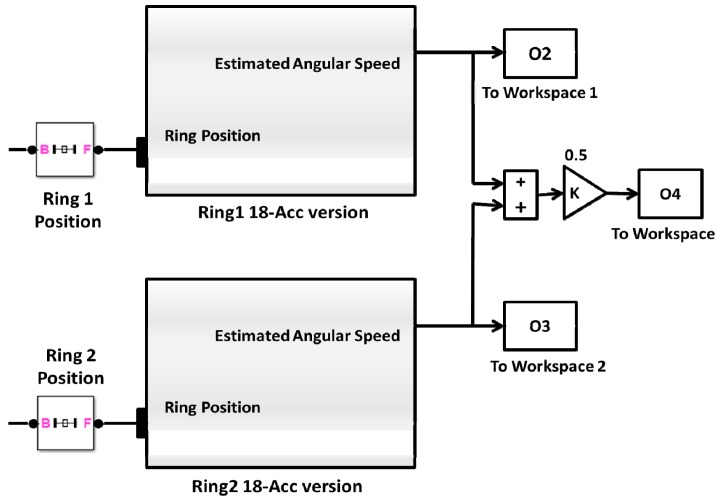
Estimate Angular Velocity using the second version of 18-accelerometers/Ring.

It is clear that this approach requires information from two rings, and, thus, demands exchange of data between the distributed rings, see Figure 3. On the other hand, the second version requires installation of accelerometers along the longitudinal axis of the aircraft, which may not be physically possible in some cases. Figure 4 shows the second version of the proposed IMU, where the additional tri-axial linear accelerometers pair is introduced onto the *X*-axis. The second version can be used separately without the need of additional rings, see Figure 5.

Using the same approach, the second version can be modeled using the following set of equations. Equation (10) can be split into state equations and measurement equations as can be found in Table 2.

(10) $$\left[\begin{array}{ccc} \frac{{\vec{A}}_{1}-{\vec{A}}_{2}}{2µ} & \frac{{\vec{A}}_{3}-{\vec{A}}_{4}}{2µ} & \frac{{\vec{A}}_{5}-{\vec{A}}_{6}}{2µ} \end{array}\right]=\left[\begin{array}{ccc} 0 & -{\dot{\Omega }}_{z} & {\dot{\Omega }}_{y}\\ {\dot{\Omega }}_{z} & 0 & -{\dot{\Omega }}_{x}\\ -{\dot{\Omega }}_{y} & {\dot{\Omega }}_{x} & 0 \end{array}\right]+\left[\begin{array}{ccc} -\Omega_{z}^{2}-\Omega_{y}^{2} & \Omega_{x}\Omega_{y} & \Omega_{x}\Omega_{z}\\ \Omega_{x}\Omega_{y} & -\Omega_{z}^{2}-\Omega_{x}^{2} & \Omega_{z}\Omega_{y}\\ \Omega_{x}\Omega_{z} & \Omega_{z}\Omega_{y} & -\Omega_{y}^{2}-\Omega_{x}^{2} \end{array}\right]$$

Since the dynamical systems presented in Tables 1 and 2 are having non-linear measurements equations, Extended Kalman Filters (EKF) can be used to implement these equations and to retrieve the angular velocity. The angular acceleration can be directly obtained by solving the state equations. Equations (11) and (12) show the matrices and the main equations used in designing the EKF for the proposed IMUs. Further details can be found in [15,16].

(11) $$\begin{array}{c} A_{i}=\left[\begin{array}{ccc} 1 & 0 & 0\\ 0 & 1 & 0\\ 0 & 0 & 1 \end{array}\right],H_{i}=2*\left[\begin{array}{ccc} 0 & \Omega_{y} & \Omega_{z}\\ \Omega_{x} & 0 & \Omega_{z}\\ \Omega_{x} & \Omega_{y} & 0 \end{array}\right],h_{i}=\left[\begin{array}{c} \Omega_{z}^{2}+\Omega_{y}^{2}\\ \Omega_{z}^{2}+\Omega_{x}^{2}\\ \Omega_{y}^{2}+\Omega_{x}^{2} \end{array}\right]\\ Q_{i}=\sigma_{Q}^{2}*I_{3\times 3},R_{i}=\sigma_{R}^{2}*I_{3\times 3}\\ B_{1}=\left[\begin{array}{c} \frac{1}{4µ}(A_{1z}-A_{2z}-A_{3y}+A_{4y})\\ \frac{-1}{2L}(A_{1z}-A_{5z})+\frac{1}{4µ}(A_{3x}-A_{4x})\\ \frac{1}{2L}\left(A_{1y}-A_{5y}\right)-\frac{1}{4µ}(A_{1x}-A_{2x}) \end{array}\right],B_{2}=\frac{1}{4\mu }\left[\begin{array}{c} \left(A_{3z}-A_{4z}-A_{5y}+A_{6y}\right)\\ \left(A_{5x}-A_{6x}-A_{1z}+A_{2z}\right)\\ \left(A_{1y}-A_{2y}-A_{3x}+A_{4x}\right) \end{array}\right] \end{array}$$

(12) $$\begin{array}{c} P_{ki}^{-}=P_{(k-1)i}^{+}+Q_{i}\\ {\hat{x}}_{ki}^{-}=A_{i}{\hat{x}}_{(k-1)i}^{+}+B_{ki}\\ K_{ki}=P_{ki}^{-}H_{ki}^{T}{\left(H_{ki}P_{ki}^{-}H_{ki}^{T}+R_{i}\right)}^{-1}\\ {\hat{x}}_{ki}^{+}={\hat{x}}_{ki}^{-}+K_{ki}[y_{ki}-h_{ki}({\hat{x}}_{ki}^{-},0)]\\ P_{ki}^{+}=(I_{3\times 3}-K_{ki}H_{ki})P_{ki}^{-} \end{array}$$

where, (*i*) denotes the Ring index, *i.e.*, *i* = 1,2, for Two-Ring case, *k* is the sample time. *I*_3×3_ is the 3 × 3 identity matrix, $\sigma_{Q}^{2}$ is the value of the process noise covariance taken to be 0.01, and $\sigma_{R}^{2}$ is the value of the measurement noise covariance taken to be 0.001.

### 3.2. Estimating the Body Linear Acceleration and the Position of the CoG Using the First Version

The angular accelerations and velocities, found previously, will be used to estimate the position of the CoG and the vehicle's linear acceleration; focus will be on Ring 1.

Referring to Equation (3), adding the accelerometers' measurements at points *P1* to *P4* will result in the following Equation:

(13) $$\frac{1}{4}({\vec{A}}_{1}+{\vec{A}}_{2}+{\vec{A}}_{3}+{\vec{A}}_{4})={\ddot{\vec{R}}}_{I}+{\ddot{\vec{R}}}_{v1}+\dot{\vec{\Omega }}\times {\vec{R}}_{v1}+2\vec{\Omega }\times {\dot{\vec{R}}}_{v1}+\vec{\Omega }\times \left(\vec{\Omega }\times {\vec{R}}_{v1}\right)-\vec{g}$$

assuming a stationary CoG, *i.e.*, ${\ddot{\vec{R}}}_{v1}={\dot{\vec{R}}}_{v1}=\vec{0}$, and a known gravitational acceleration *g⃑*, then Equation (13) can be given as follows:

$$\vec{f}={\ddot{\vec{R}}}_{I}+B{\vec{R}}_{v1}$$

where:

(14) $$\begin{array}{c} \vec{f}=\frac{1}{4}\left({\vec{A}}_{1}+{\vec{A}}_{2}+{\vec{A}}_{3}+{\vec{A}}_{4}\right)+\vec{g}\\ B=\left[\begin{array}{ccc} b_{11} & b_{12} & b_{13}\\ b_{21} & b_{22} & b_{23}\\ b_{31} & b_{32} & b_{33} \end{array}\right]=\left[\begin{array}{ccc} 0 & -{\dot{\Omega }}_{z} & {\dot{\Omega }}_{y}\\ {\dot{\Omega }}_{z} & 0 & -{\dot{\Omega }}_{x}\\ -{\dot{\Omega }}_{y} & {\dot{\Omega }}_{x} & 0 \end{array}\right]+\left[\begin{array}{ccc} -\Omega_{z}^{2}-\Omega_{y}^{2} & \Omega_{x}\Omega_{y} & \Omega_{x}\Omega_{z}\\ \Omega_{x}\Omega_{y} & -\Omega_{z}^{2}-\Omega_{x}^{2} & \Omega_{z}\Omega_{y}\\ \Omega_{x}\Omega_{z} & \Omega_{z}\Omega_{y} & -\Omega_{y}^{2}-\Omega_{x}^{2} \end{array}\right] \end{array}$$

Equation (14) can be solved using a QR-Decomposition based Weighted Recursive Least Squares (QR-D based WRLS) with Forgetting Factor (FF) and covariance matrix resetting threshold (TH). Using a Forgetting Factor helps in enhancing the performance when tracking time-varying systems, however sometimes it may cause instability of the identification method if its value is not selected properly. Another way to enhance the performance is by introducing a conditional covariance matrix resetting procedure by which the tracking capability of such method is drastically increased [17]. Actually, the method used here was incorporated with this procedure taking the trace of the covariance matrix as the condition upon which the decision to reset the covariance matrix is made.

The identification problem is solved as follows:

Rearrange (14) in the form of:

$$\vec{f}=D\vec{x}$$

where:

(15) $$\begin{array}{c} \vec{x}={\left[r_{v1x},r_{v1y},r_{v1z},a_{x},a_{y},a_{z}\right]}^{T}\\ D=\left[\begin{array}{ccc} b_{11} & b_{12} & \begin{array}{ccc} b_{13} & 1 & \begin{array}{cc} 0 & 0 \end{array} \end{array}\\ b_{21} & b_{22} & \begin{array}{ccc} b_{23} & 0 & \begin{array}{cc} 1 & 0 \end{array} \end{array}\\ b_{31} & b_{32} & \begin{array}{ccc} b_{33} & 0 & \begin{array}{cc} 0 & 1 \end{array} \end{array} \end{array}\right] \end{array}$$

Equation (15) is implemented within a QR-D based WRLS scheme as follows:

1. Find the QR-Decomposition of matrix *D* to enhance its condition number. This can be done using different methods, such as Householder or Givens Rotations. In this work, the (*qr*) MATLAB function was used.
2. Update the Regression Expression found in Equation (15) as follows:
  (16) $$\begin{array}{c} \vec{f}=D\vec{x}=Q*R*\vec{x}\to Q^{T}*\vec{f}=R*\vec{x}\\ Q^{T}*\vec{f}=R*\vec{x}\to \vec{w}=R*\vec{x} \end{array}$$
3. Now, use the modified Regression Expression in a WRLS scheme as follows [18]:
  (17) $$\begin{array}{c} K_{ki}=\frac{P_{\left(k-1\right)i}R_{ki}^{T}}{FF+\text{trace}\left(R_{ki}P_{\left(k-1\right)i}R_{ki}^{T}\right)}\\ {\vec{e}}_{ki}\left(k\right)=\vec{w}\left(k\right)-R_{ki}{\vec{\hat{x}}}_{i}\left(k-1\right)\\ {\vec{\hat{x}}}_{ki}\left(k\right)={\vec{\hat{x}}}_{ki}\left(k-1\right)+K_{ki}{\vec{e}}_{ki}\left(k\right)\\ P_{ki}=\frac{1}{FF}(I-K_{ki}R_{ki})P_{\left(k-1\right)i} \end{array}$$
  The following expression will help in keeping the covariance matrix positive [16]:
  $${\text{P}}_{ki}=0.5*({\text{P}}_{ki}^{\text{T}}+{\text{P}}_{ki})$$
  where, (*i*) denotes the Ring index, *i.e.*, *i* = 1,2 , for Two-Ring case, and *k* is the sample time.
4. Now, check the trace of the covariance matrix as follows:
  (18) $$\begin{array}{c} \text{if}\left(\text{Trace}\left(P_{ki}\right)>\text{TH}\right)\text{then}\\ P_{ki}=\beta *I_{6\times 6} \end{array}$$
  where, *I*_6×6_ is the 6 × 6 identity matrix, and the used parameters' values were found through trial and error and were as follows: *FF* = 0.75 = *TH* = 10^5^ and *β* = 1000.

The same calculations are done independently for all rings. As in the case of estimated angular velocities and accelerations, see [9], the best estimate of the position of CoG with respect to say, Ring 1, is given for the Two-Ring case by:

(19) $${\overset{⌢}{\bar{R}}}_{v1}=\frac{1}{2}\left({\overset{⌢}{\vec{R}}}_{v11}+{\overset{⌢}{\vec{R}}}_{v12}\right)=\frac{1}{2}({\overset{⌢}{\vec{R}}}_{v1}+{\overset{⌢}{\vec{R}}}_{v2}+L\vec{i})$$

where, ${\overset{⌢}{\vec{R}}}_{v11}={\overset{⌢}{\vec{R}}}_{v1}$ is the estimation of the CoG position relative to the first Ring calculated using the measurements of the first ring, while ${\overset{⌢}{\vec{R}}}_{v12}={\overset{⌢}{\vec{R}}}_{v2}+L\vec{i}$ is the estimation of the CoG position relative to the first Ring calculated using the measurements of the second Ring, and *L* is the distance between the two rings in the *X* direction. Finding the position of CoG relative to the air vehicle datum is also possible, provided that the equations are updated accordingly.

### 3.3. Estimating the Body Acceleration and the Position of the CoG Using the Second Version

The same procedure used with the first version can be used here with the modification:

(20) $$\vec{f}=({\vec{A}}_{1}+{\vec{A}}_{2}+{\vec{A}}_{3}+{\vec{A}}_{4}+{\vec{A}}_{5}+{\vec{A}}_{6})/6+\vec{g}$$

**Figure 6:**
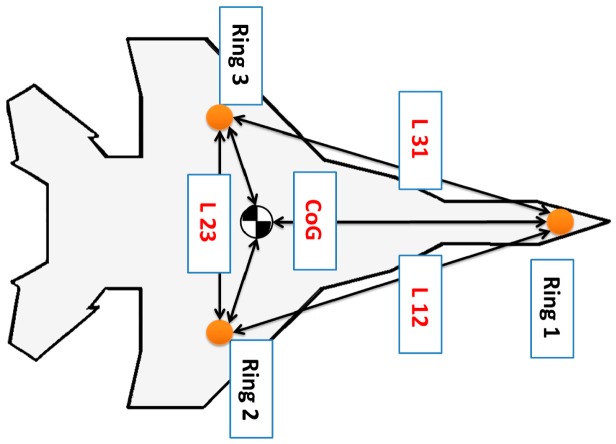
A jet fighter with three-Ring configuration using the second version.

Using more rings can improve further the reliability of the measurements and the estimation of the navigation parameters along with the position of CoG. For the second version, each ring can be placed anywhere within the airframe. All that is needed, in this case, is the relative distances between the rings (*L*_12_,*L*_23_,*L*_31_) and the position of each ring with respect to the vehicle datum. Figure 6 reflects the flexibility of installing the second version within an airframe.

In the next section, the simulation results of the two IMU versions are presented. The usage of SimMechanics Library found in Simulink facilitates the simulation of the proposed IMUs.

## 4. Simulation Results and Discussion

In this part, the simulation results of the proposed IMUs are presented. The SimMechanics Library has been used to model an arbitrary object in 3D space subjected to various Forces and Torques. SimMechanics blocks were used since they give more freedom in the design process as well as different situations can be investigated, such as misalignments and disorientation in the accelerometers, when they are assembled to form a ring.

Since the proposed IMU depends heavily on the accelerometers used, a 3-Axis model for an accelerometer was built and completely parameterized to make it easily reconfigurable and reusable with minimum modification. The main objective of this section is to show how the proposed IMUs can be used to estimate the position of the CoG.

## 3-Axis Accelerometer Modeling

Practical accelerometers' measurements do involve noise and also are subjected to faults; hence an independent noise source for each axis in the same accelerometer is implemented. Faults were modeled to be one of the following: None, no-outputs, and wrong-output which each axis in the same accelerometer can have.

It is worth noting that the bias and cross coupling effect, and other errors, usually encountered in real 3-axis accelerometers were not included. Faults are not simulated in this work, see [9] for more information.

**Figure 7:**
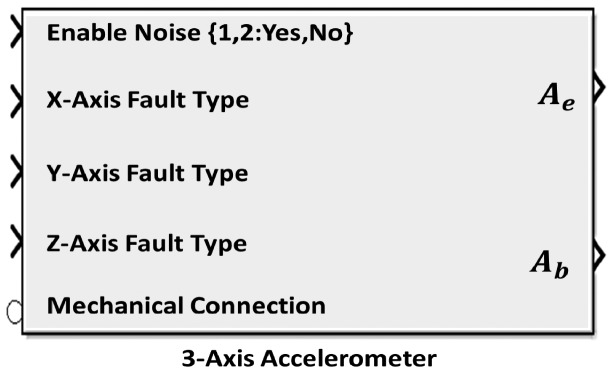
3-axis accelerometer model. Acceleration measurements relative to the World (Ae) and body (Ab) coordinates.

**Figure 8:**
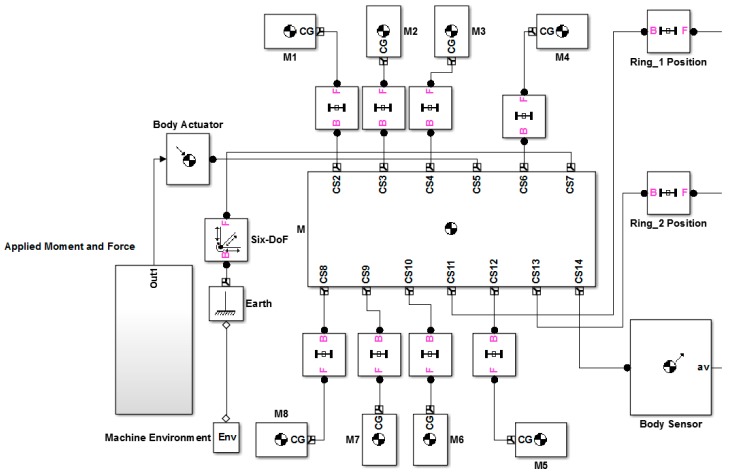
Composite rigid body SimMechanics Machine.

**Figure 9:**
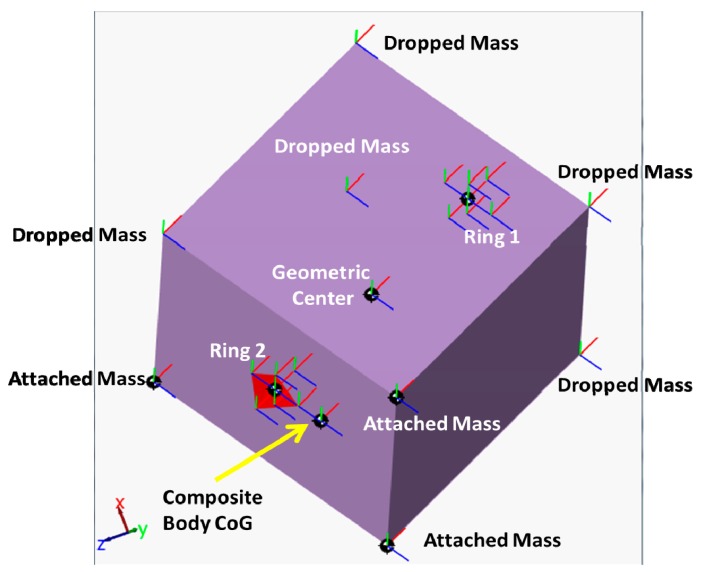
SimMechanics visualization of the composite rigid body given in the example at T = 60 s.

The accelerometer model, shown in Figure 7, was used in the simulation to form the two rings. Figure 8 shows a SimMechanics machine that resembles a composite rigid body in the form of cube whose mass is (0.5 kg) and the length of its side is (1 m) with (1 kg) added masses at each corner subjected to force and moment acting at its equivalent CoG and it is allowed to move in 6-DOF motion in the space where no gravity force is active, *i.e.*, *g⃑* = 0⃑. Figure 9 clearly represents this composite body shape.

It can be seen from Equation (14) that the *B* matrix depends totally on the rotational motion of the body. So, it is a must to have sufficient rotational motion to obtain a good estimation of the position of CoG using this approach. Next, the different versions of the proposed IMU are simulated. The simulation results reveal the performance of each version and highlight some points to be discussed afterword.

**Figure 10:**
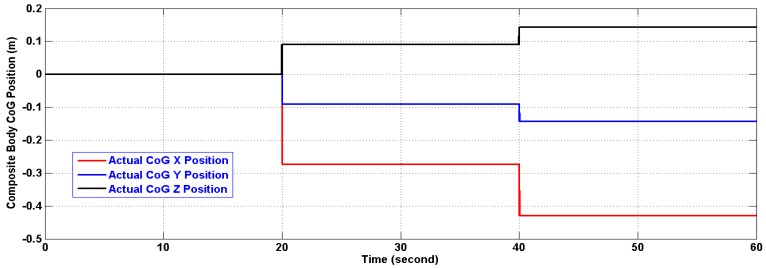
Composite body CoG Position (m) relative to the Cube Geometric Center (0,0,0).

The CoG of the composite body was found using the following equation:

(21) $$\text{CoG}=\frac{\sum_{1}^{d}m_{i}{\vec{r}}_{i}}{\sum_{1}^{d}m_{i}}$$

where (*d*) is the total number of additional masses, that is 8, (*m_i_*) is the value of the added mass taken here to be (1 kg), and (*r⃑_i_*) is its position relative to the cube geometric center in meters. The position of the composite body CoG, relative to the cube geometric center (0,0,0), was forced to change by dropping a number of added masses at the end of each interval, an interval equals 20 s in simulation time. The effect is resembled by abrupt changes in its position as can be seen in Figure 10. The new composite inertia is calculated by SimMechanics and the resulting dynamic equation is solved internally. The two Rings were located at (0.5,0,0) meters and (−0.5,0,0) meters respectively. Table 3 shows the schedule used in dropping the additional masses and the resulting CoG position.

The dimensions and weights used in the simulation example are typical in small Unmanned Arial Vehicles (UAV) applications, although the example itself is for a generic body. However, it is also possible to estimate the CoG position in true-sized aerial vehicles using the proposed approach provided that sufficient angular motion is available to render the parameters observable.

The addition of a third Ring (or more) can be easily incorporated using the presented analysis in this paper.

The simulation results of the first version are shown next assuming ideal sensor network and by taking (*L* = 1 m, μ = 0.1 m)

**Figure 11:**
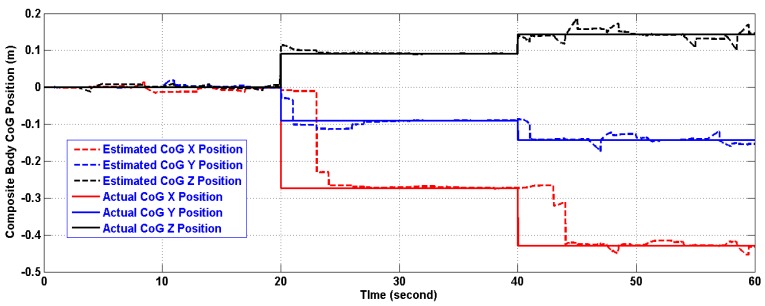
Estimation of the composite body CoG position (m) using the first version in Two-Ring Configuration.

**Figure 12:**
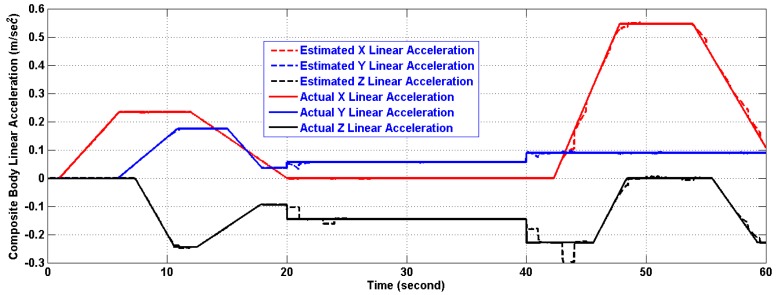
Estimation of the composite body acceleration (m/s^2^) using the first version in Two-Ring Configuration.

**Figure 13:**
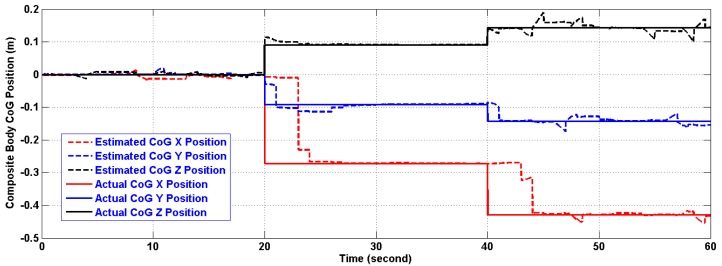
Estimation of the composite body CoG position (m) using the second version in Two-Ring Configuration.

**Figure 14:**
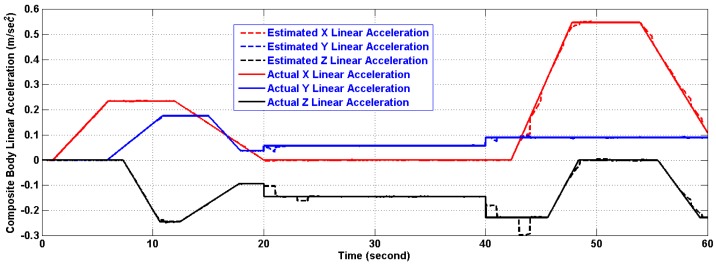
Estimation of the composite body acceleration (m/s^2^) using the second version in Two-Ring Configuration.

In the period of (30–40) s when the linear acceleration is constant and the CoG is not moving from its new position, the algorithm estimates both of them with reasonable accuracy. However, further testing for the algorithm under time varying acceleration in period (50–60) s showed an excellent tracking of linear acceleration and the CoG with reasonable accuracy. The artifacts of Figures 11 and 13 will fade out in the steady state. As a matter of fact, the artifacts happen at the corner change of the linear acceleration as depicted in Figures 12 and 14. The gaps in CoG position estimation as found in Figures 11 and 13 were due to the fact of insufficient angular motion, *i.e.*, angular velocity and/or acceleration. This was designed to reflect the importance of the angular motion existence on the performance of the presented algorithm.

The simulation results of the second version are shown next and by taking (μ = 0.1 m).

Tables 4 and 5 show the errors in linear acceleration and CoG position estimations using the two versions after 10 seconds from the first CoG position change. This was designed to reflect the fact that further investigation of the identification technique along with a quantitative description of the minimum angular motion and a better filter are needed which will be a subject of future work.

Another thing that can be observed from Tables 4 and 5 is the fact that the performance of the QR-D based WRLS will be affected by CoG change so robust and adaptive techniques should be investigated. In those techniques, the relation between the filter used to retrieve the angular motion and the identification technique must be considered; because the performance of the latter will be affected by the performance of the former as can be read from Equation (14).

Although the simulation results and the Maximum Percent Error (MPE)/Normalized Mean Square Error (NMSE) values do not reflect a huge difference in performance between the proposed IMUs, it is still obvious that each version provides attractive features that the other may not have. Table 6 presents a brief comparison between the two IMU versions. However, sensitivity analysis will show the effect of the relation between the Rings separation; *i.e.*, (*L*), and (μ) on the first version overall performance.

Moreover, the more accelerometers' measurements used the better the estimation will be and that can be done by using additional Rings (IMUs) and then fuse the estimation results obtained from the individual Rings. In the previous context, this was done using simply the average value although more sophisticated techniques can be used to fuse those estimations.

The MPE and NMSE are given by Equations (22) and (23) respectively.

(22) $$\text{MPE}=\max\left(\frac{\text{True}-\text{Estimate}}{\text{True}}\right)*100\%$$

(23) $$\text{NMSE}=\frac{\sum_{i=1}^{N}{\left(\text{True}(i)-\text{Estimate}(i)\right)}^{2}}{N*\underset{i}{\text{max}}(\text{abs}{\left(\text{True}(i)\right)}^{2}}$$

where, *N* is the number of samples.

Another approach to consider when using the proposed IMUs is the Centralized estimation where all the accelerometers' measurements are fed into one central estimation unit that utilizes all the measurements and obtain better estimation results.

In the first version, robust estimation of the angular velocities requires measurements from at least two Rings. Acquisition of the measurements from the distributed sensors to a centralized processing unit may require additional considerations for the sensor network capacity and reliability.

Another important consideration is the minimum angular motion needed to ensure reliable estimation of the CoG. Table 7 shows a descriptive plan that helps in estimating the position of CoG using the proposed IMUs. This plan was obtained by examining the nature of the *B* matrix given by Equation (14). This plan can be implemented in both manned and unmanned aerial vehicles. Table 7 can be used to investigate the relation between the air vehicle's trajectory and the CoG estimation using the proposed IMUs. The table indicates that to estimate the change in the position along any of the vehicle axis, we need angular (rotational) motion components on at least one of the other two axes.

A final point to mention is that the quality of CoG position estimation is affected by the filter used to retrieve the angular velocity from the accelerometers' measurements, the identification technique used, and the available angular motion.

## 5. Conclusions

The paper presented an improved IMU with a method for tracking the changes in the position of the Center of Gravity of an air vehicle due to fuel consumption or changes in payload. The proposed IMU is based on an all-accelerometers design for low cost and reliable design. The proposed IMU comes in two versions to accommodate installations constraints. Table 6 provided additional information on the differences between the two versions. The simulation example showed how the proposed IMU can be used in estimating the position of the Center of Gravity, and showed the importance of the presence of the angular motion to have robust estimation. The Maximum Percent Absolute Error was less than 1 percent in both versions in estimating the position of the Center of Gravity, where the second version showed a slightly better performance. The performance of the first version is also influenced by the Rings' separation; *i.e.*, (L), as well as (μ).

New measurement models can be obtained from Equations (9) and (10) to explore other Kalman filter techniques to estimate the angular velocities. Similarly, other on-line systems identification methods could also be explored for estimation of the position of the Center of Gravity. Since the proposed IMU estimates the linear/angular accelerations, the angular velocities, and the position of Center of Gravity, it can then be used in building an improved Inertial Navigation System. The available accelerometers' measurements can also be used within the proposed IMU in fault detection and isolation, and, at the same time, to increase the precision of the measurements. The proposed IMU has many potential applications, since knowledge of the position of the Center of Gravity is essential for proper calculation of the various aerodynamic forces and torques on an aircraft or missile body, for selection of the proper control strategy, and for ensuring vehicle stability and effective guidance.

## Figures and Tables

**Table 1.** Equations of first version.

State Equations (First Version)	Measurements Equations
${\dot{\Omega }}_{x}=\frac{1}{4µ}(A_{1z}-A_{2z}-A_{3y}+A_{4y})$	$\frac{1}{L}\left(A_{5x}-A_{1x}\right)=\Omega_{z}^{2}+\Omega_{y}^{2}$
${\dot{\Omega }}_{y}=\frac{-1}{2L}(A_{1z}-A_{5z})+\frac{1}{4µ}(A_{3x}-A_{4x})$	$\frac{1}{2µ}\left(A_{2y}-A_{1y}\right)=\Omega_{z}^{2}+\Omega_{x}^{2}$
${\dot{\Omega }}_{z}=\frac{1}{2L}\left(A_{1y}-A_{5y}\right)-\frac{1}{4µ}(A_{1x}-A_{2x})$	$\frac{1}{2µ}\left(A_{4z}-A_{3z}\right)=\Omega_{y}^{2}+\Omega_{x}^{2}$

**Table 2:** Equations of second version.

State Equations (Second Version)	Measurements Equations
${\dot{\Omega }}_{x}=\frac{1}{4µ}(A_{3z}-A_{4z}-A_{5y}+A_{6y})$	$\frac{1}{2µ}\left(A_{2x}-A_{1x}\right)=\Omega_{z}^{2}+\Omega_{y}^{2}$
${\dot{\Omega }}_{y}=\frac{1}{4µ}(A_{5x}-A_{6x}-A_{1z}+A_{2z})$	$\frac{1}{2µ}\left(A_{4y}-A_{3y}\right)=\Omega_{z}^{2}+\Omega_{x}^{2}$
${\dot{\Omega }}_{z}=\frac{1}{4µ}(A_{1y}-A_{2y}-A_{3x}+A_{4x})$	$\frac{1}{2µ}\left(A_{6z}-A_{5z}\right)=\Omega_{y}^{2}+\Omega_{x}^{2}$

**Table 3:** Simulation schedule.

Total Mass (kg)	Composite Body CoG Position (m)	Dropped Mass Position (m)	Time Interval (s)
8.5	(0,0,0)	-	0–20
		(0.5,0.5,0.5)	
5.5	(−0.2727,−0.0909,0.0909)	(0.5,−0.5,−0.5)	20–40
		(0.5,0.5,−0.5)	
3.5	(−0.4286,−0.1429,0.1429)	(0.5,−0.5,0.5)	40–60
		(−0.5,0.5,−0.5)	

**Table 4:** CoG Position and linear acceleration estimations MPE in the period (30–40 s).

Axis	CoG Position		Linear Acceleration	
	
	First Version	Second Version	First Version	Second Version
*X*	0.2572	0.1643	--*	--*
*Y*	0.4069	0.1508	1.0212	0.8527
*Z*	0.6265	0.3978	0.4451	0.4620

* The MPE of the *X*-axis linear acceleration was undefined in this interval; since its true value was zero

**Table 5:** CoG Position and linear acceleration estimations NMSE in the period (30–40 s).

Axis	CoG Position		Linear Acceleration	
	
	First Version	Second Version	First Version	Second Version
*X*	2.4252e−06	1.0008e−06	--*	--*
*Y*	1.3992e−05	1.3390e−05	6.3191e−06	5.1146e−06
*Z*	1.5306e−05	4.4980e−06	2.7849e−06	2.3104e−06

* The NMSE of the *X*-axis linear acceleration was undefined in this interval; since its true value was zero.

**Table 6:** Brief comparison between the two IMU versions.

Property	First Version	Second Version
Size requirements	2-D	3-D
Self-contained	No	Yes
Affected by Rings separation	Yes	No
Installation requirements	Vehicle *X*-axis	Any where

**Table 7:** Angular motions for proper estimation of the CoG.

CoG Change	Ω*_x_*	Ω*_y_*	Ω*_z_*
*X*-Axis			
*Y*-Axis			
*Z*-Axis			
*X*-*Y* Plane			
*X*-*Z* Plane			
*Y*-*Z* Plane			
*X*-*Y*-*Z*			
*X*-*Y*-*Z*			
*X*-*Y*-*Z*